# Optimised Implant Selection Using Digital Volume Tomography (DVT) Osteodensitometry

**DOI:** 10.1155/2022/2470524

**Published:** 2022-02-14

**Authors:** Manfred Nilius, Jörg Winterhoff

**Affiliations:** ^1^Niliusklinik, Londoner Bogen 6, D-44269 Dortmund, Germany; ^2^Universitätsklinikum, Carl Gustav Carus, Technische Universität Dresden, Fakultät für Zahn, Mund-und Kieferheilkunde, Dresden, Germany

## Abstract

**Purpose:**

The density of the jaw bone has an inhomogeneous distribution and, even within partial volumes, varies more or less strongly from the size of typical implants. Consequently, the primary stability of implants using conventional techniques can only ever be determined postimplantation. The aim of the present case report is to present digital volume tomography (DVT) osteodensitometry as a procedure for assessing the primary stability preimplantation and to evaluate its benefits.

**Methods:**

An orthopantomogram (OPT) and vertical sections, including bone density measurement, were obtained from a DVT in the course of preimplantological diagnosis. Conventional implant planning and densitometric simulation were performed on this basis.

**Results:**

Densitometric simulation enabled assessment of the bone density at the interface to the implant body preimplantation. This procedure provides not only an overall value (averaged density) but also allocation of bone areas with different densities to the adjacent areas on the implant surface. This then allows the implant with the best possible macroscopic geometry and optimum position to be selected, with the aim of maximising primary stability. In the present case, the maximum torques during insertion confirmed the densitometric values recorded in advance.

**Conclusion:**

DVT osteodensitometry enables selection of an implant optimised to bone density distribution. At the same time, the point at which loading occurs can already be defined at the planning stage, thanks to the predictable primary stability. A standard integration of DVT osteodensitometry in the algorithms of implant planning software thus seems desirable.

## 1. Introduction

There are a variety of technologies using Cone Beam-Computer Tomography (CB-CT) scan and CAD/CAM to measure implant stability: cutting resistance analysis (CRA), reverse torque test (RTT), resonance frequency analysis (RFA), and impact hammer method [1].

The standard methods in implantology—torque measurement during insertion [2, 3] and implant stability quotient (ISQ) [4–6] for evaluating the primary stability—have proven successful in implant planning. They have the disadvantage, however, of providing the required information only after insertion is complete. But reliable assessment of bone quality for evaluating the possible moment of loading already at the planning stage would be useful for the clinician, so that the timing of the treatment sequence is predictable and can be communicated in a patient-oriented manner.

The use of DVT osteodensitometry opens up the possibility of a predictive method [7–11]. With the aid of DVT, not only the bone density can be evaluated, but also the expected bone contact with the implant can be shown—differentiated according to areas of different quality/thickness. This allows the expected primary stability already to be predicted in preoperative diagnosis. The dental surgeon can decide individually in advance, whether prosthetic immediate loading is possible or whether a delayed restoration is to be preferred, to reduce the risk of implant failure in the early stage. Bone condensing or augmentative measures can also be evaluated in this way [7, 8].

The following case shows an example of a densitometrically planned implantation from the planning stage to prosthetic rehabilitation and to the 3-year follow-up.

## 2. Case Presentation

**Figure 1:**
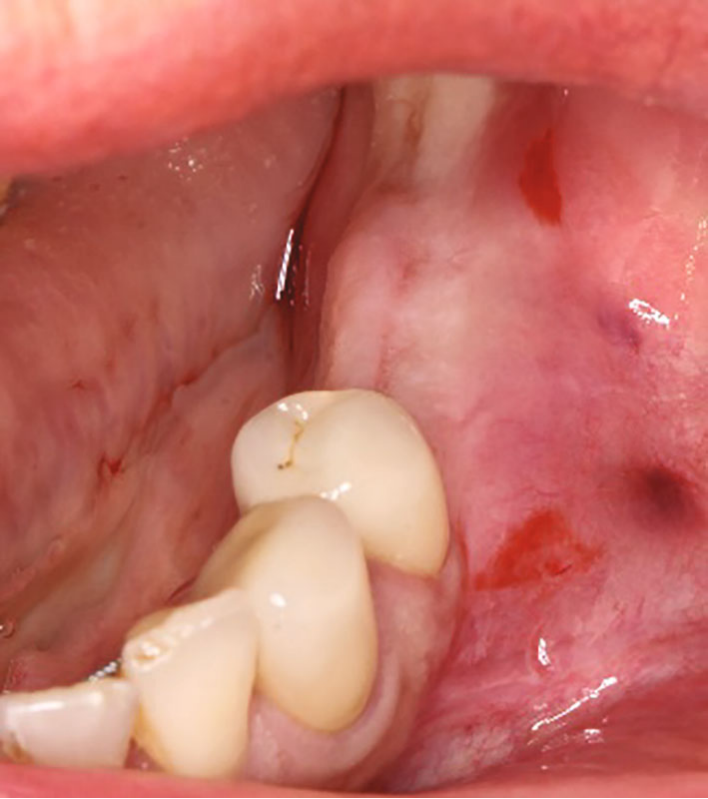
The initial clinical situation.

A 55-year-old female patient presented with the request for a fixed restoration in the left mandible. She was in good general condition and had no systemic preexisting diseases. The periodontal indices were inconspicuous. There was a slight narrowness in the lower jaw front. Previous patient's dental history was an abutment loss of tooth 36 with shortening of the existing bridge to the distal of the crowned tooth 34. In this area, there was evidence of a completely healed region with moderate atrophy of the alveolar ridge (Figure 1).

Following comprehensive patient history, diagnosis, and consultation, the patient decided in favour of an implant-borne restoration in the form of all-ceramic crowns. Likewise, the insufficient crowns on 33 and 34, which were on teeth worth preserving, should also be replaced.

### 2.1. Preimplantological Diagnosis and Planning

The aim of preimplantological diagnosis was to ensure the best possible selection of implants for the local bone with regard to the expected primary stability and thus the planning of the time of loading.

**Figure 2:**
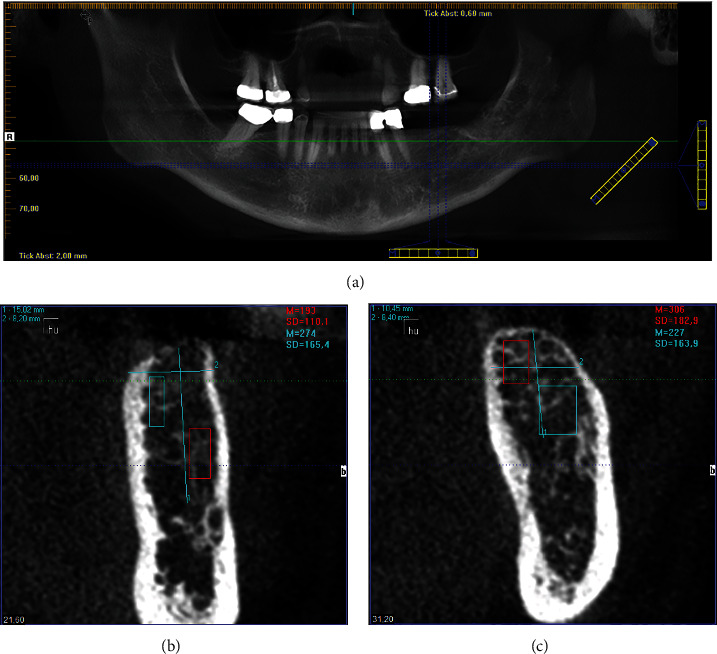
An OPT and a vertical section in the region of the implants to be inserted were derived from the DVT.

Intraoral scans (3Shape Trios 3 Basic Pod, Software: 3Shape Implant Studio Version 20.1.1, Copenhagen/Denmark) of the initial situation were taken and a DVT created (CB-CT: KaVo 3D-eXam ConeBeam XG; software: eXam-VisionQ, version 1.9.3.13, both by Kaltenbach & Voigt Dental GmbH, Biberach/Riss, Germany). From these, a level-optimised OPT and a vertical section in the region of implants to be placed were also generated using Hounsfield unit measurement (Figures 2(a)–2(c)).

**Figure 3:**
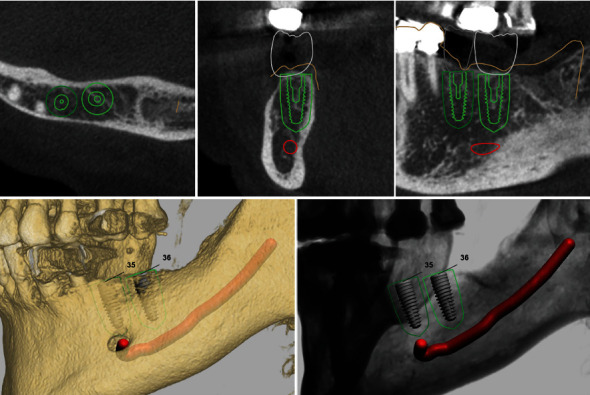
Screenshot from the guided surgery planning.

After importing the DVT data, including Hounsfield values, in an implant planning software (Implant Studio, 3Shape, Copenhagen), the densitometric-supported planning for the guided surgery process was completed (Figure 3).

In addition to the usual determination of implant position, length, and diameter, a densitometric-based simulation could also be created using this data/software combination. This provides visualisation of which parts of the implant body come into contact with areas of different bone density.

Apart from limited and restricted flexible parameters, such as implant length and diameter due to anatomical conditions, this additional information now allows the macrostructure of the implant body to be evaluated in relation to its effect on the (primary) stability in the bone site and selected accordingly.

**Figure 4:**
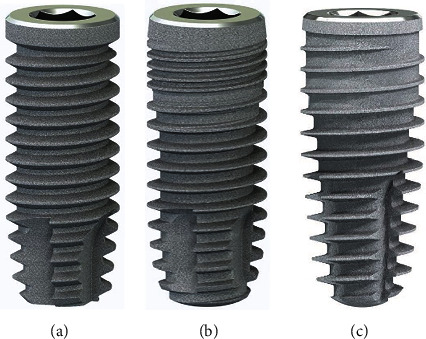
(a) SICace (cylindrical). (b) SICmax (mainly cylindrical). (c) SICtapered (conical).

For this, it was also an advantage to have an implant system available that provides various implant bodies with the same instrumentarium and the same prosthetic interface (SIC invent, Basel, Switzerland). The system selected in the present case allows selection between three different types of implant body (SICace, SICmax, SICtapered, Figures 4(a)–4(c)) using the same drill instrumentarium and protocol.

**Figure 5:**
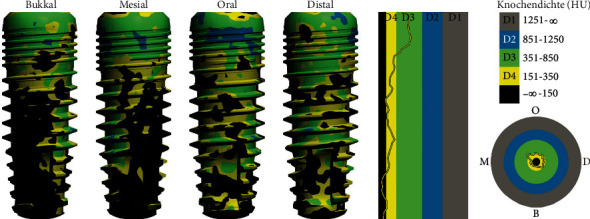
Example: simulation with mainly cylindrical implant (SICmax).

**Figure 6:**
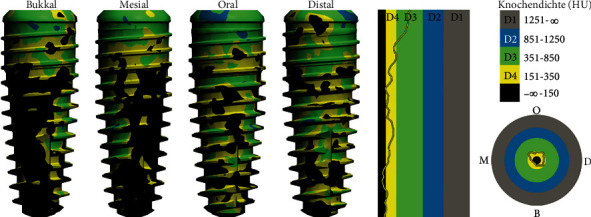
Simulation with conical implant (SICtapered).

We performed densitometric simulation for the different implant bodies of the system and ultimately decided in favour of the conical implant (SICtapered), as there was mainly soft bone present towards the buccal side at the implant position (Figures 5 and 6). The defined compression effect of the conical implant seemed the sensible option to us in this case (Figure 6).

Immediate loading was excluded due to the predominantly D3/D4 bone density, and unloaded healing was planned. According to planning, the guided surgery template was fabricated in the dental laboratory using the implant software, and the drill protocol to be used was printed and provided for treatment.

### 2.2. Implantation and Prosthetic Restoration

**Figure 7:**
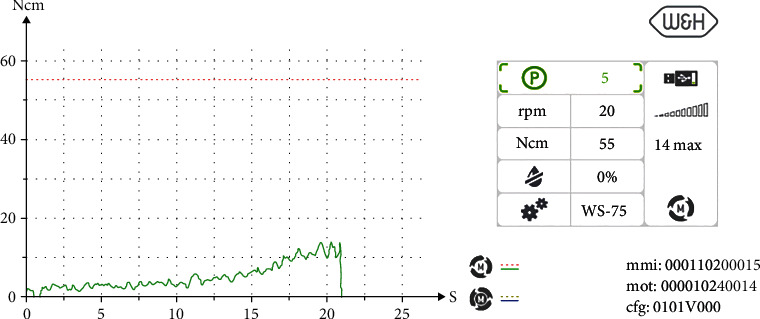
Insertion torque curve 35.

**Figure 8:**
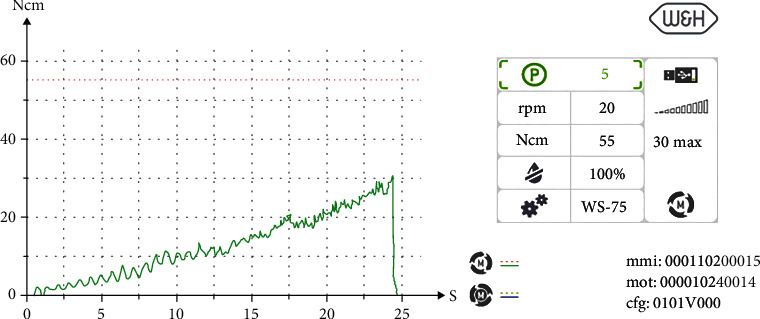
Insertion torque curve 36.

Treatment was performed using electronically controlled intraosseous anaesthesia (Quicksleeper 5, Dental HiTec, Gronau, Germany) with approximately 0.8 ml Ultracain DS-Forte osteocentral in the implant insertion region [12]. This avoided the use of block anaesthesia and associated risk, while also reducing the volume of local anaesthetic. Thanks to the planning reliability of the DVT-based guided surgery technique, a flapless procedure could be performed to ensure minimum traumatisation of the surrounding hard and soft tissue. Preparation of the implant site was guided, i.e., using the template with the corresponding instrumentarium (SIC Guided Surgery, SIC invent, Basel). Preparation and implant insertion proceeded without any complications, and the implants were inserted using maximum torques of 14 Ncm (35) and 30 Ncm (36) (Figures 7 and 8). This corresponded to the expected values from the densitometry of minimum compression of the spongiosa, without a final peak through the crestal compact bone, and supported the planning of unloaded healing.

**Figure 9:**
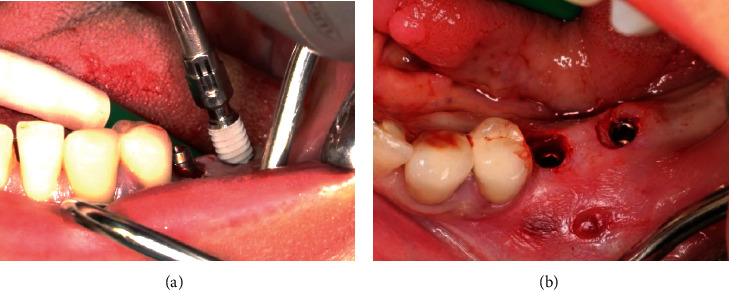
The implants were inserted in regions 35 and 36.

**Figure 10:**
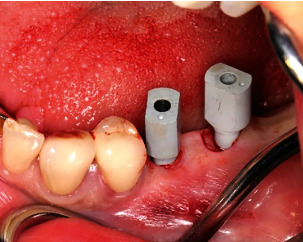
Digital impression posts (SIC Scan Adapter for CAD/CAM abutments and milling blanks) with buccal alignment of the point for the immediate postoperative scan.

The intraoral scan for the long-term temporary restoration (LTT) was taken immediately after insertion (Figures 9(a), 9(b), and 10).

**Figure 11:**
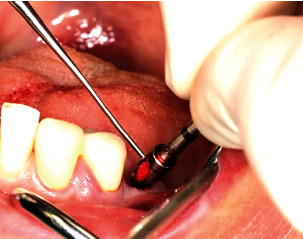
Subcrestal relocation of local bone (bone chips from the crestal cutter, SIC Crestal Cutter) on the implant shoulder following insertion of the implant cover screw.

**Figure 12:**
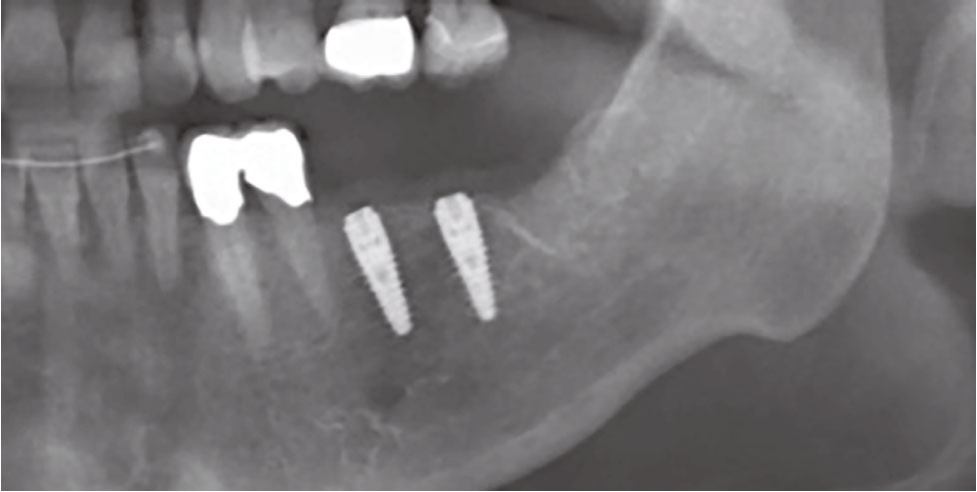
X-ray check after insertion.

Filling of small bone defects in the cervical areas of the implant using intraoperatively harvested host bone chips and closure using sutures (Profimed 3/0, medipac, Kilkis, Greece) completed the treatment (Figures 11 and 12).

The patient explicitly expressed her satisfaction with the treatment procedure at the check-up on the following day and reported discreet initial wound pain.

**Figure 13:**
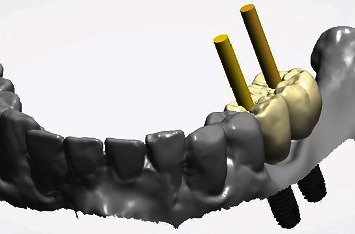
Verification of the implant axes (yellow pins) for implants 35 and 36 and comparison with the prosthetic digital crown planning.

Based on the intraoral scan from the day of treatment and the DVT data, which was matched in the implant planning software, the LTT was then digitally planned in the form of two single crowns (Figure 13). The temporary restorations were fabricated using a multilayer polymethylmethacrylate (PMMA) composite milling blank (Copra Temp Symphony A2-PMMA-Blank, Whitepeaks, dental solutions GmbH) on titanium- (Ti-) bonding bases (SIC bonding bases CAD/CAM) with the final ZrO_2_ abutments (DD cube ONE, white, Dental Direkt, Spenge, Germany), which are also custom milled.

Ti bases with PMMA, with the required transgingival emergence profile for contouring the soft tissue, were fabricated as gingival formers following implant exposure and fitted five months after insertion. Both implants proved to be optimally osseointegrated and exhibited ISQ values of 72 and 75 (Penguin RFA Instrument Kit, P-I Brånemark, Askim, Sweden/SIC invent, Basel). X-ray images and sound on percussion affirmed the clinical findings.

**Figure 14:**
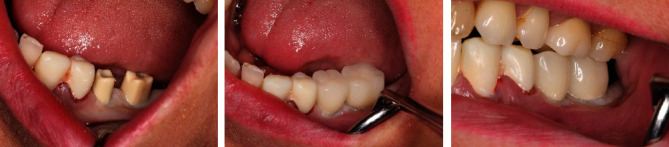
Fitting the long-term temporary restorations.

**Figure 15:**
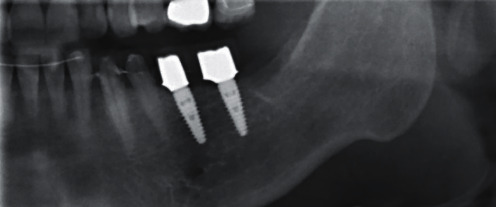
X-ray check during LTT fitting.

Due to the accompanying aligner treatment (Invisalign, Align Technology, Tempe, Arizona, USA) desired during the course of treatment, the temporary restoration was only fitted two months later (Figure 14). The customised final ZrO_2_ abutments on Ti bonding bases (DD cube ONE, white, Dental Direkt, Spenge) were finally screwed in position using the recommended torque of 20 Ncm for fitting. The insufficient crowns on teeth 33 and 34, which were due to be renewed, were removed, and the teeth built up at the same appointment. The opportunity was also used to take an X-ray (Figure 15).

After final preparation of teeth 33 and 34, a renewed intraoral mandibular scan was created for fabricating the final restoration following completion of the aligner treatment. In addition to the prepared teeth 33 and 34, the scan also included the 35 and 36 abutments.

**Figure 16:**
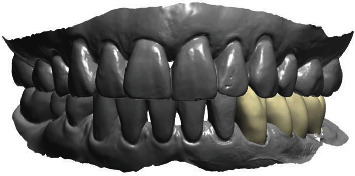
The design of the final crowns in the CAD process.

The final crowns were designed virtually in the dental laboratory in the CAD process (Figure 16) and then milled from ZrO_2_ (DD cube ONE, white, Dental Direkt, Spenge) and characterised (MiYO, Jensen, Metzingen, Germany).

**Figure 17:**
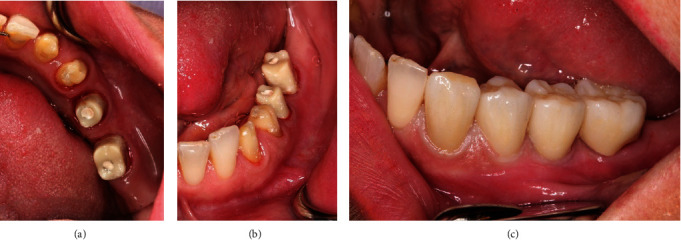
Fitting of the final crowns using dual-curing cement.

After a total treatment time of approximately 18 months, including aligner treatment, the final restoration of the support zone could be completed using crowns on the teeth and implants 33, 34, 35, and 36 (Figures 17(a)–17(c)). The crowns were fitted using dual-curing luting composite (Variolink Esthetic DC neutral, Ivoclar Vivadent, Schaan, Liechtenstein). The patient was highly satisfied with the aesthetics and function.

**Figure 18:**
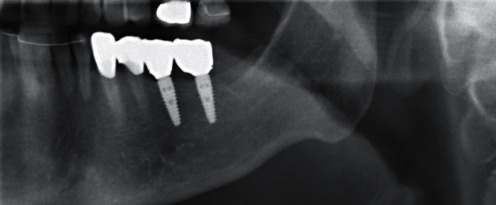
X-ray check at the crown fitting.

A final X-ray check was also taken at the same appointment (Figure 18).

**Figure 19:**
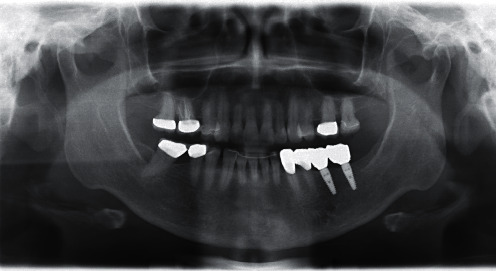
X-ray check 3 years after implant insertion.

The patient still comes regularly for a check-up. The latest check OPG three years after implant insertion shows an unchanged stable bone level (Figure 19). The clinical functionality and soft tissue situation are also unchanged, and the patient is highly satisfied with them.

## 3. Discussion

The present case demonstrates how implant planning and treatment using modern digital methods for diagnosis, planning, and fabrication lead to treatment reliability and predictable results. DVT-based planning, with osteodensitometry analysis and insertion simulation, plays a decisive role in this. It allows case-by-case selection and optimum positioning of the implants. In this way, it is possible for the first time, using objective data and their imaging, to be able to evaluate osseointegration conditions already at the planning stage and to make the correct decision for the time at which to load the implants.

Any changes to the planning, such as the required aligner treatment after beginning treatment in the present case, could be implemented in the digital workflow without problem based on the intraoral scan. Flapless technique can be used when the minimal keratinized tissue height of 2 mm has remained [13].

Any prosthetic adjustments to the masticatory function due to the long-term temporary restorations could also be undertaken. The selected approach proved flexible, including with the decision for a specific type of implant body (cylindrical, mainly cylindrical or conical). The decision can still even be made at the moment of insertion after preparation is complete. In contrast to parallel forms, the tapered form selected in the present case still allows compression of the bone to be varied by slightly adjusting the insertion depth to create better primary stability [14].

The next stage in the ongoing development of implantological planning software could consequently be in the inclusion of DVT-based osteodensitometry in the algorithm. The programme would then make far-reaching suggestions for implant positions, insertion (conventional or flapless), and suitable type of implant body. This could possibly be further optimised using artificial intelligence—just like the suggestions of the planning software.

It is self-evident that treatment decisions are ultimately made by the implantologists, if necessary following a consultation meeting together with the patient. It would seem preferable that this can be explained even more clearly regarding possible treatment options using imaging of the further advanced software. “Implantology must become simpler”! If this dogma is followed, we expect in future informed patients who, following a visit to an implantological centre, are highly motivated and look forward to their implantological rehabilitation, thanks to appropriate explanation and reliably predicted treatment schedule.

Note: some photographs in this case were unspecifically, for clarification of an example of a treatment procedure, already published in the magazine *EDI Journal* within the framework of a study into the reliability of a certain implant design (SICtapered) [15].

## Data Availability

The data used to support the findings of this study are included within the article.
